# Data pipeline for real-time energy consumption data management and prediction

**DOI:** 10.3389/fdata.2024.1308236

**Published:** 2024-03-13

**Authors:** Jeonghwan Im, Jaekyu Lee, Somin Lee, Hyuk-Yoon Kwon

**Affiliations:** ^1^Graduate School of Data Science, Seoul National University of Science and Technology, Seoul, Republic of Korea; ^2^Department of Global Technology Management, Seoul National University of Science and Technology, Seoul, Republic of Korea

**Keywords:** energy consumption, MLOps-centric data pipeline, time-series forecasting, real-time data pipeline, scalable pipeline

## Abstract

With the increasing utilization of data in various industries and applications, constructing an efficient data pipeline has become crucial. In this study, we propose a machine learning operations-centric data pipeline specifically designed for an energy consumption management system. This pipeline seamlessly integrates the machine learning model with real-time data management and prediction capabilities. The overall architecture of our proposed pipeline comprises several key components, including Kafka, InfluxDB, Telegraf, Zookeeper, and Grafana. To enable accurate energy consumption predictions, we adopt two time-series prediction models, long short-term memory (LSTM), and seasonal autoregressive integrated moving average (SARIMA). Our analysis reveals a clear trade-off between speed and accuracy, where SARIMA exhibits faster model learning time while LSTM outperforms SARIMA in prediction accuracy. To validate the effectiveness of our pipeline, we measure the overall processing time by optimizing the configuration of Telegraf, which directly impacts the load in the pipeline. The results are promising, as our pipeline achieves an average end-to-end processing time of only 0.39 s for handling 10,000 data records and an impressive 1.26 s when scaling up to 100,000 records. This indicates 30.69–90.88 times faster processing compared to the existing Python-based approach. Additionally, when the number of records increases by ten times, the increased overhead is reduced by 3.07 times. This verifies that the proposed pipeline exhibits an efficient and scalable structure suitable for real-time environments.

## 1 Introduction

With the growing utilization of data in various industries and applications, the construction of efficient data pipelines becomes paramount. These pipelines are responsible for designing the entire process from data collection to data services, ensuring smooth and effective data management. In particular, real-time pipelines have the capability to dynamically collect and process data from Internet of Things (IoT) sensors, enabling them to provide instantaneous and up-to-date services (Gogineni et al., 2015; Rathore et al., 2015; Kalsoom et al., 2020). As a result, real-time data pipelines have gained significant traction in various industries (Gogineni et al., 2015; Rathore et al., 2015; Kalsoom et al., 2020). However, conventional Python scripts commonly used for MLOps often exhibit relatively long processing times in the overall pipeline. Moreover, they demonstrate a significant increase in overhead as the number of records grows. Therefore, the establishment of a real-time data pipeline is necessary to address these challenges.

This study focuses on energy consumption management systems, aiming to design an efficient data pipeline that covers the entire process from data collection to time series prediction models. The critical importance of energy management has led many countries to adopt energy consumption prediction models, which strive to optimize their performance (Ekonomou, 2010; Shapi et al., 2021). Machine learning (ML) has emerged as a powerful approach to tackling complex challenges across various industries, delivering effective solutions. To ensure the seamless operation of ML models, all interconnected system components, including data collection and management, must work in harmony, not just for the ML model itself (Sculley et al., 2014). In this context, machine learning operations (MLOps) have arisen as a viable solution to design an efficient system framework.

In this study, we introduce a specialized MLOps-focused framework that seamlessly integrates the ML model with the data pipeline for real-time energy consumption data management and prediction. In the framework, energy consumption data is collected from IoT sensors, undergoes preprocessing, and is then stored in databases. To deal with real-time collected data without loss, Kafka is employed for data ingestion, and Telegraf consumes the data from Kafka and feeds them to the database. Finally, the time-series prediction models, long short-term memory (LSTM) and seasonal autoregressive integrated moving average (SARIMA), are adopted.

To evaluate the performance of the models, we conducted a comprehensive comparison using a substantial dataset of electric power consumption. The results highlighted the trade-off between speed and accuracy. SARIMA exhibited a remarkable advantage in model learning time, making it suitable for scenarios with strict real-time requirements, while LSTM outperformed SARIMA in prediction accuracy.

To validate the effects of the pipeline, we measured the processing time of the overall pipeline by optimizing the configuration of Telegraf. In particular, we showed that our pipeline has an average end-to-end processing time of only 0.39 s to deal with 10,000 data records at a time in the pipeline and only 1.26 s when expanding it to 100,000 records. This indicates 30.69–90.88 times faster processing compared to the existing Python-based approach. Additionally, when the number of records increases by ten times, the increased overhead is reduced by 3.07 times. This verifies that the proposed pipeline exhibits an efficient and scalable structure suitable for real-time environments.

## 2 Related work

### 2.1 Data pipeline

Fu and Soman (2021) introduced a data infrastructure tailored for handling real-time data generated by end-users in Uber. The system employed Apache Kafka for real-time data ingestion and Apache Flink for stream processing. By utilizing these tools, they were able to effectively process and analyze the continuous flow of real-time data, enabling prompt responses to user actions and improving overall system performance. Similarly, Syafrudin et al. (2017) proposed a real-time data processing framework specifically designed for sustainability in the manufacturing sector. Their framework integrated Apache Kafka for detecting data transmitted from sensors, Apache Storm for real-time data processing, and MongoDB for storing and managing the processed data. With this setup, they were able to ensure timely data processing and storage, facilitating efficient decision-making and resource management in the manufacturing context. Both studies showcase the significance of real-time data processing frameworks in various industries. By leveraging powerful tools such as Apache Kafka, Apache Flink, Apache Storm, and MongoDB, these frameworks can handle large volumes of data in real-time, leading to improved operational efficiency, better decision-making, and enhanced user experiences.

### 2.2 Time series prediction model

Initially, various machine learning methodologies, such as autoregressive integrated moving average(ARIMA)-based statistical methods (Amjady, 2001; Chujai et al., 2013; Fard and Akbari-Zadeh, 2014), support vector machine (SVM) (Mohandes, 2002; Fan et al., 2016), and decision tree (Huang et al., 2016; Mayrink and Hippert, 2016), were commonly employed for predicting energy consumption. These traditional techniques provided valuable insights and paved the way for energy consumption forecasting.

However, in recent years, the field of energy consumption prediction has witnessed a significant shift toward the application of deep learning models. Artificial neural networks (ANN) (Gajowniczek and Zabkowski, 2014; Massana et al., 2015), especially LSTM models (Kong et al., 2017; Kim et al., 2018), which are capable of learning temporary gating functions, have shown remarkable performance in this domain. LSTM's ability to handle sequential data and capture long-term dependencies makes it well-suited for time series prediction tasks. Therefore, there have been various research efforts that utilize LSTM for predicting individual household energy consumption (Yan et al., 2019), gas consumption (Laib et al., 2019), and long-term energy consumption (Wang et al., 2020) have been proposed.

Additionally, convolutional neural networks (CNN) (Amarasinghe et al., 2017; Koprinska et al., 2018) have also demonstrated outstanding results in energy consumption prediction. CNNs excel at extracting local correlations between power spectrums, making them highly effective in analyzing spatial and temporal patterns in energy consumption data.

These deep learning models have revolutionized energy consumption prediction, offering improved accuracy and more robust predictions compared to traditional machine learning methods. However, in practice, we need to consider their excessive costs in an environment equipped with embedded devices such as IoT sensors.

## 3 Background

### 3.1 InfluxDB

InfluxDB is a renowned open-source time-series database, developed by InfluxData. Its primary focus lies in efficiently storing time-series data, offering fast processing and high availability for both write and read operations. This makes it a popular choice for applications that heavily rely on time information, such as IoT sensors, smart factories, operation monitoring, and real-time analysis. InfluxDB's versatility, performance, and ease of use make it a prominent tool in the time-series data management landscape.

### 3.2 Kafka

Kafka is a powerful open-source distributed event streaming platform developed by Apache. Its primary purpose is to capture data in real-time from various sources, including databases and sensors, in the form of a continuous stream of events. The architecture of Kafka revolves around three key components: producers, consumers, and brokers. Producers are responsible for publishing or writing the events into Kafka. Consumers, on the other hand, subscribe to the events and read them from Kafka. Brokers play a crucial role as mediators between producers and consumers. They efficiently manage and group the events into topics, which are essentially named streams of data.

### 3.3 Zookeeper

Zookeeper is a valuable open-source coordination service developed by Apache. Its main purpose is to simplify the implementation of various tasks in distributed environments, including synchronization and master node selection. In the context of Kafka, Zookeeper plays a crucial role in monitoring the health of the servers and storing the status of topics for both producing and consuming data. When new topics are created, Zookeeper keeps track of their status and ensures that consumers and producers are informed about the existence of these new topics. Zookeeper works closely with Kafka brokers to maintain a reliable and up-to-date system. It notifies consumers and producers about the creation of new Kafka brokers and any potential failures that may occur.

### 3.4 Telegraf

Telegraf is an open-source plugin developed by InfluxData, designed to efficiently collect and deliver events from databases, systems, and IoT sensors. One of the significant advantages of Telegraf is its versatility, as it can be seamlessly integrated into various server applications like InfluxDB and Kafka. This flexibility allows Telegraf to be easily incorporated into existing data flows, enabling users to leverage its capabilities without major disruptions to their current setups. By acting as a powerful data collection and delivery tool, Telegraf contributes to enhancing data management, analysis, and real-time processing.

### 3.5 Grafana

Grafana is a powerful open-source toolkit developed by Grafana Labs, specifically designed to offer a comprehensive dashboard for visualizing time-series data. One of Grafana's key strengths lies in its compatibility with multiple databases, including ElasticSearch, InfluxDB, and PostgreSQL. This enables users to seamlessly connect their data sources and create insightful visualizations. Grafana serves as an invaluable tool for data visualization and monitoring, offering a user-friendly interface and seamless integration with diverse data sources and notification systems.

## 4 Proposed framework

### 4.1 Data ingestion pipeline

**Figure 1:**
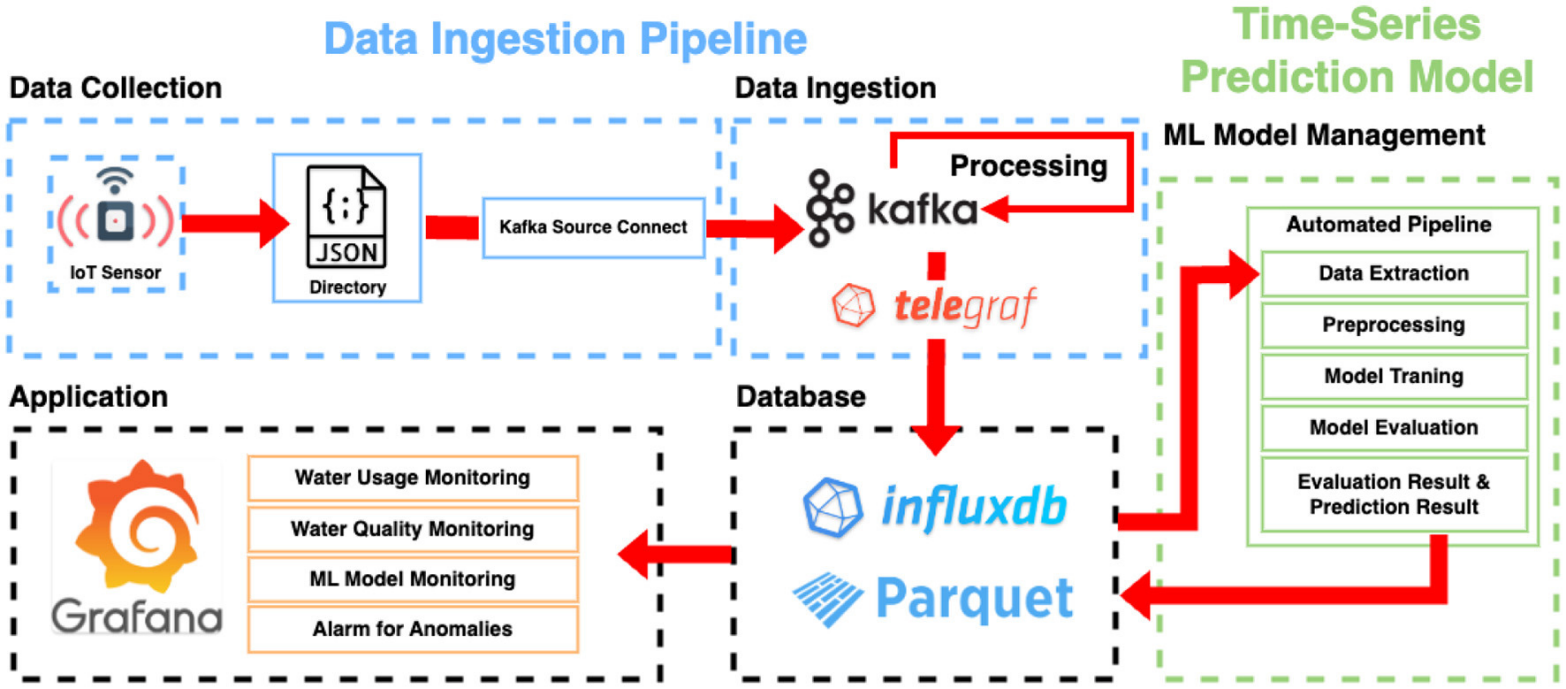
The overall architecture of the proposed framework.

Figure 1 illustrates the overall architecture of the proposed framework. The data ingestion pipeline is highlighted in blue, while the time-series prediction model is highlighted in green. The data ingestion pipeline consists of two main parts: data collection and data ingestion. The data collection, which is the initial stage of the architecture, involves gathering energy consumption data from IoT sensors. The collected data is transmitted to the central server. Subsequently, the Kafka source connector monitors the specified path and sends the data to the Kafka brokers for further processing. Once the data is in Kafka, it undergoes several processing steps. The processing steps occur before the data is transmitted to the main database, InfluxDB, after being consumed by Telegraf from Kafka. These steps consist of (1) parsing, (2) transformation, and (3) reconstruction. The parsing step involves decoding the collected sensor data into a usable format. The raw sensor data is encoded as a nested JSON in a single string, containing a timestamp, identifier, and sensor measurement values. To insert the encoded string data into the database, it is decoded to the individual attributes. The transformation step converts the decoded attributes from the parsing step into attribute types defined in the database schema. For example, it performs the conversion of a timestamp, initially formatted as a string, into a Unix timestamp defined in the database. The reconstruction step involves restructuring the original data for efficient data storage. Storing a nested JSON value directly in the database can lead to a degradation in query performance. Therefore, the nested data is appropriately restructured into a more manageable format in the database. For instance, sensor data incoming at the district level is reconstructed at the household level. These processes enhance the overall stability of the system, enabling efficient data storage and queries.

To avoid an excessive accumulation of data in InfluxDB that could result in a decline in query performance and resource wastage, data older than a certain time threshold is compressed in the Apache Parquet format. These compressed files can be accessed and visualized in Grafana.

### 4.2 Time-series prediction model

To begin the prediction process, data is retrieved from both the Parquet files and InfluxDB. The retrieved data then undergoes extraction and preprocessing steps to prepare it for model training.

In the extraction step, the sensor data utilized for model training is extracted from the entire dataset^1^ and is passed through the process of model training. We read the target data used for training from Parquet files and InfluxDB. The read data is loaded into a Pandas dataframe, facilitating the training process. In the preprocessing step, we perform preprocessing on the sensor data to prepare it for model training. Three main processes are executed. First, we fill out the missing values. Sensor data often contains missing values, which can lead to degraded performance during model training and prediction. We replace missing values with the most recent value. Second, we adjust the time unit for model prediction. Before conducting model training and prediction, it is essential to adjust the time unit of the collected data to that for model training and prediction. In this step, we aggregate the collected sensor data to the time unit for prediction. For instance, if the collected data is at a 1-min interval and the prediction unit is at a 1-h interval, the collected data is aggregated into 1-h intervals. Third, we structure the original data into sliding window units. For time series prediction models, a sliding window configuration is crucial for training and prediction. This process involves grouping the continuous time series data for each time point into an array format. For example, if there is time series data with a time sequence from 1 to 15, and the sliding window size is 10, the sliding windows would be structured as 1–10, 2–11, ..., and 6–15.

The chosen prediction models, SARIMA (Valipour, 2015) and LSTM (Hochreiter and Schmidhuber, 1997), are trained using preprocessed data. SARIMA incorporates seasonal components in addition to ARIMA, often used to identify or impose regular patterns in time series data, especially in data types with periodicity. Temperatures are representative features with periodicity. Because energy consumption is significantly correlated with temperature, SARIMA is suitable for considering such seasonality. LSTM has the ability to retain information for a period and is beneficial for processing a long sequence. Because time series data is a prominent example of sequence data, LSTM is a suitable candidate for predicting time series data.

To monitor the models' ongoing performance, the evaluation metrics and prediction results are stored in the database. This enables easy access to the historical performance of the models and facilitates analysis and decision-making. The prediction process is applied to predict energy consumption in real-time, allowing for timely and informed management decisions. To capture recent trends in the data and account for changes in the system, the entire prediction process is periodically automated. This periodic automation ensures that the models remain up-to-date and can adapt to dynamic changes in the energy consumption data. By integrating SARIMA and LSTM models within the MLOps-based framework, the system can take advantage of both statistical time-series modeling and deep learning-based techniques. This comprehensive approach aims to optimize prediction efficiency while targeting real-time service requirements in energy management.

## 5 Performance evaluation

### 5.1 Experimental environments

For the experiments, we used a server equipped with Intel Xeon Silver 4210R CPU 2.40 GHz, 32 GB of memory, and Nvidia RTX A5000, where Ubuntu OS is installed. We use the following versions of the software: Python 3.6.9, Kafka 5.5.0, Zookeeper 3.4.9, InfluxDB 1.8.2, Telegraf, 1.19, and Grafana 7.2.0.

### 5.2 Experimental datasets and methods

The dataset (Hebrail and Berard, 2012) used in this study was collected with a one-minute sampling rate over a period between December 2006 and November 2010, encompassing a total of 47 months. It is a multivariate time-series data consisting of six independent variables, such as electrical quantities and sub-metering values, along with a numerical dependent variable, i.e., global active power. The dataset contains a total of 2,075,259 observations. During preprocessing, the data was resampled to an hourly frequency from the original one-minute sampling rate. The first 3 years of data (36 months) were used for model training, while the remaining data (11 months) were reserved for validation purposes.

To measure the prediction accuracy of the models, two types of metrics were employed: Mean Absolute Error (MAE) as shown in Equation (1), and Root Mean Squared Error (RMSE) as shown in Equation (2). MAE measures the average magnitude of the errors, *e*_*i*_, in a set of predictions, without considering their direction. It is the average over the test sample of the absolute differences between prediction and actual observation where all individual differences have equal weight. It is a suitable indicator when the loss increases linearly. RMSE represents the standard deviation of the prediction errors, *e*_*i*_. Since the error is squared, the larger the error, the higher the weight accordingly is reflected.


(1) $$\begin{array}{l} MAE=\frac{1}{n}\overset{n}{\underset{i=1}{\sum }}|e_{i}| \end{array}$$

(2) $$\begin{array}{l} RMSE=\sqrt{\frac{1}{n}\overset{n}{\underset{i=1}{\sum }}e_{i}^{2}} \end{array}$$

For the hyperparameters of LSTM, ADAM (Kingma and Ba, 2014) was chosen as the optimizer, with a learning rate of 0.001, and MAE was used as the loss function. ADAM is a learning optimization algorithm that dynamically adjusts the learning rates for individual parameters, striking a balance between the global and local optima. It offers the advantage of relatively swift learning rates and excellent optimization performance. As for the SARIMA model, the ARIMA order was set as (1,1,1), and the seasonal order was set as (1,0,1,24). These parameter settings were determined based on the lowest Akaike Information Criterion (AIC) (Burnham and Anderson, 2004), which is a measure of the relative quality of statistical models for a given set of data. AIC represents the similarity between actual data and predicted data, which tends to be higher when there are more independent variables used in the estimation. As for AIC, it increases when unnecessary parameters are added to improve fitness, so a lower AIC suggests a better model with fewer unnecessary parameters. It is important to note that the results may vary depending on the chosen hyperparameters for both SARIMA and LSTM models. The presented hyperparameter settings were specifically chosen for this study to provide insights into the prediction accuracy of the models on the resampled data.

### 5.3 Experimental results for a prediction model

**Figure 2:**
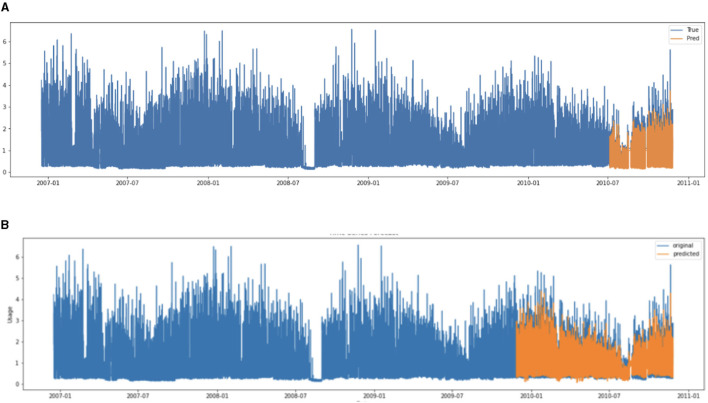
Visualization of predicted results of LSTM and SARIMA. **(A)** LSTM and **(B)** SARIMA.

Figure 2 shows the predicted results of the energy consumption using SARIMA and LSTM. Table 1 presents the predicted results of SARIMA and LSTM. Overall, in terms of accuracy, LSTM outperforms SARIMA, but it is essential to highlight that SARIMA still demonstrates acceptable accuracy. In regard to model learning time, SARIMA shows a significant advantage over LSTM, being ~30.58 times faster. This indicates that SARIMA could be a more desirable choice when the prediction model needs to run on resource-constrained IoT devices or when frequent updates of the prediction model are required. On the other hand, LSTM proves to be more desirable in scenarios where more accurate predictions are needed and computational resources are less constrained, such as server environments.

**Table 1:** Comparing model learning time and accuracy.

	**Model learning time**	**RMSE**	**MAE**
SARIMA	1 min 19 s	0.5103	0.3592
LSTM	40 min 16 s	0.0825	0.1084

### 5.4 Experimental results for a data pipeline

**Figure 3:**
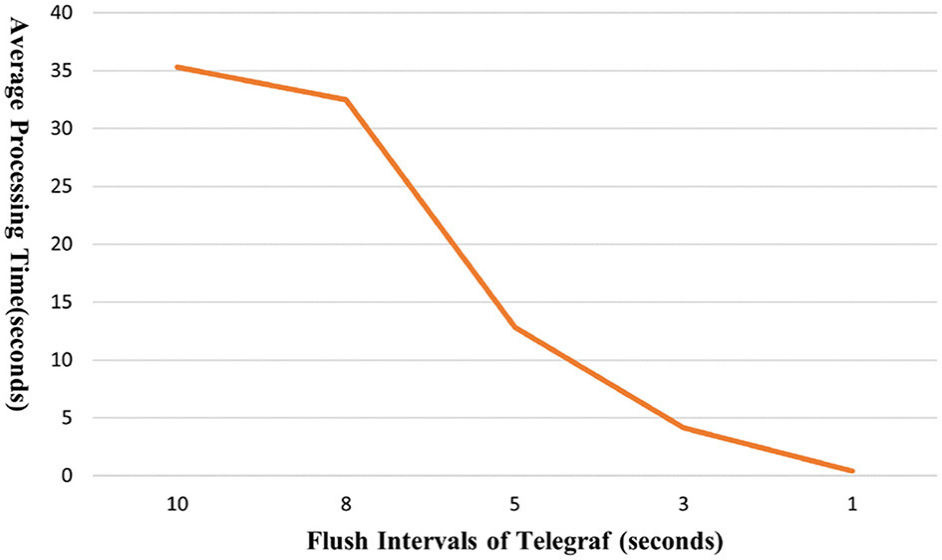
Average processing time from data collection to database.

To optimize the performance of the entire pipeline, Telegraf plays a crucial role in consuming data from Kafka. To assess the scalability of the data pipeline, we conducted tests by generating artificial data at a rate of 500 records per second, randomly selected from the real-world dataset. To observe the overall processing time, we adjusted the flush interval of Telegraf from 10 to 1 s, loading 10,000 data records. Figure 3 shows the results of the overall processing time for the entire data pipeline of the proposed framework. The results showed that the smallest flush interval (1 s) yielded the fastest performance. Specifically, with a flush interval of 10 s, the average end-to-end processing time was 35.33 s. This time decreased significantly to 12.84 and 0.39 s when the flush interval was reduced to 5 and 1 s, respectively. It is worth noting that these improvements reached a saturation point. In this setup, even with an increased data load of 100,000 records, the processing time remained impressively low at only 1.26 s. This demonstrates the framework's ability to efficiently handle large volumes of data. For comparative experiments to show the effectiveness of the proposed method, we implement a Python-based script that performs the same function. The Python script performs data loading and processing in the same manner as the proposed pipeline. Specifically, it loads and parses json files, performs transformation and reconstruction steps, and then stores them in InfluxDB. The experiments using the Python script with the same data show an average processing time of 11.57 s for 10,000 records and 114.51 s for 100,000 records, respectively. This indicates that the proposed pipeline processes data 30.69 times faster for 10,000 records and 90.88 times faster for 100,000 records compared to the Python script, respectively. That is, with a tenfold increase in the number of records, the average processing time for the Python script increases by 9.90 times, while the proposed pipeline increases by only 3.23 times. This shows that the proposed pipeline is a highly efficient pipeline suitable for real-time environments, demonstrating superior scalability.

## 6 Conclusions

In this study, we presented a comprehensive data pipeline that facilitates real-time data collection from IoT sensors, storage in databases, and time-series prediction models. Through the experiments, we showed the trade-off between the accuracy and efficiency of the ML-based prediction models under the proposed pipeline. Our pipeline showed 30.69–90.88 times faster processing than the existing Python-based approach. Additionally, when the number of records increased by ten times, the increased overhead was reduced by 3.07 times. This verifies that the proposed pipeline exhibits an efficient and scalable structure suitable for real-time environments. Given the limited research efforts in this domain, our proposed framework showcases its effectiveness in energy consumption management.

Based on the findings and insights gained from this study, we have identified two future research directions for improving the current framework. First, we need to enhance the coordination and optimization of the components within the pipeline to handle larger workloads more efficiently. As the system scales and deals with increased data volumes, it becomes crucial to ensure seamless interactions among different components and maximize resource utilization, particularly for real-time applications. Second, in this study, SARIMA and LSTM, which are simple yet effective predictive models considering the real-time nature of the proposed pipeline, were utilized. The proposed pipeline can also be extended with state-of-the-art predictive models such as MTAD-GAT (Zhao et al., 2020) and TFT (Lim et al., 2021). However, not only prediction performance but also computational overheads such as model training/inference time and resource usage should be considered. By addressing the trade-off, we aim to choose the most appropriate predictive model for a given environment.

## Data availability statement

The original contributions presented in the study are included in the article/supplementary material, further inquiries can be directed to the corresponding author.

## Author contributions

JI: Data curation, Formal analysis, Investigation, Methodology, Resources, Visualization, Writing – original draft. JL: Data curation, Investigation, Methodology, Resources, Writing – original draft. SL: Data curation, Investigation, Methodology, Resources, Writing – original draft. H-YK: Conceptualization, Formal analysis, Funding acquisition, Investigation, Methodology, Project administration, Supervision, Validation, Writing – review & editing.

## Footnotes

^1^Machine Learning Repository: http://archive.ics.uci.edu/dataset/235/individual+household+electric+power+consumption.
